# Colecistitis aguda acalculosa asociada a hepatitis A en adolescente

**DOI:** 10.1016/j.aprim.2025.103410

**Published:** 2025-11-15

**Authors:** Ecatherine Rodríguez Urteaga, Inés Osiniri Kippes, Luis Ortiz-González

**Affiliations:** a Centro de salud Dos de Mayo, Móstoles, Madrid, España; b Clínica Bofill, Girona, España; c Departamento de Ciencias Biomédicas, Facultad de Medicina y Ciencias de la Salud, Universidad de Extremadura, Badajoz, España

La colecistitis aguda acalculosa (CAA) es una entidad clínica caracterizada por la inflamación de la vesícula biliar, sin cálculos biliares. A diferencia de la colecistitis aguda litiásica, cuya etiología está bien establecida, la CAA presenta una patogenia multifactorial. En el ámbito pediátrico, la enfermedad vesicular es poco frecuente en comparación con los adultos. Sin embargo, cuando se presenta inflamación de la vesícula biliar en niños, la CAA representa la forma más común, constituyendo entre el 50 y el 70% de los casos descritos. En este grupo etario, la causa suele ser predominantemente infecciosa, a diferencia de los adultos, donde los factores críticos como el ayuno prolongado, el uso de nutrición parenteral o las enfermedades sistémicas graves son más frecuentes^1^, ^2^.

Dentro de los agentes infecciosos implicados, además de las bacterias y algunos parásitos, se han documentado múltiples casos de CAA asociada a infecciones virales. Particularmente, se ha observado una asociación significativa con el virus de Epstein-Barr (VEB) y con el virus de la hepatitis A (VHA)^2^, ^3^. El VHA, aunque típicamente cursa de manera autolimitada y suele provocar infecciones asintomáticas o formas leves de hepatitis aguda, en raras ocasiones suele estar implicado en la etiopatogenia de CAA en niños.

La fisiopatología de la CAA en el contexto de la hepatitis viral aguda sigue siendo un área de incertidumbre en la literatura, debido a la escasez de estudios concluyentes que expliquen los mecanismos precisos implicados. Diversas hipótesis han sido propuestas para intentar esclarecer este fenómeno. Entre los factores más frecuentemente sugeridos se encuentran la hipoalbuminemia, la inflamación hepática con extensión local hacia estructuras vecinas, y la hipertensión portal, los cuales podrían contribuir al desarrollo de edema en la pared de la vesícula biliar, un hallazgo común en estos pacientes. Además, algunos estudios han sugerido que el VHA podría tener un tropismo particular hacia el epitelio biliar. Esta afirmación se sustenta en la detección de antígenos virales en células epiteliales de los conductos biliares mediante técnicas inmunohistoquímicas, lo cual indicaría un posible efecto citopático directo del virus sobre dichas estructuras. La interacción viral con el epitelio biliar podría favorecer la aparición de colestasis intrahepática y contribuir al desarrollo de CAA en pacientes con hepatitis aguda^4^.

El diagnóstico de la CAA en este contexto se establece fundamentalmente a través de la correlación de hallazgos ecográficos característicos (engrosamiento de la pared vesicular sin presencia de litiasis) con pruebas serológicas que confirman la infección viral. No obstante, debido a que la etiología no es de origen bacteriano ni obstructivo, el tratamiento suele ser conservador. En la mayoría de los casos, la resolución espontánea de la inflamación vesicular ocurre tras la recuperación del cuadro viral agudo.

En este contexto, se presenta el caso de una adolescente de 12 años con dolor en epigastrio e hipocondrio derecho, astenia, fiebre y vómitos de 72 h de evolución. La ecografía clínica mostró engrosamiento mural vesicular en «capas de cebolla» de hasta 8 mm (normal < 3 mm), sin cálculos ni barro biliar, y sin flujo en Doppler color (fig. 1 y vídeo). Fue remitida al hospital, donde se objetivaron hipertransaminasemia, hiperbilirrubinemia y serología positiva para el VHA, diagnosticándose CAA asociada al VHA. El tratamiento conservador logró la resolución completa clínica, analítica y ecográfica (fig. 2).

**Figura 1:**
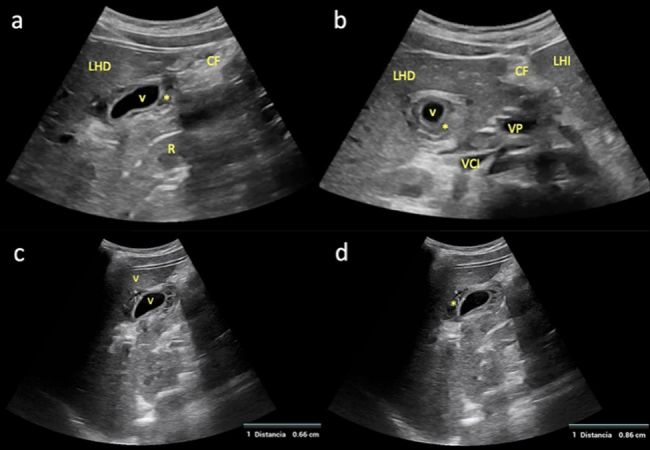
a) Corte longitudinal paramedial del abdomen a nivel de hipocondrio derecho con sonda convex (1,5-4,5 MHz), en el que se objetiva una sección sagital de la vesícula biliar (VB) con pared engrosada (*), sugerente de CAA; b) Corte transversal del abdomen con la misma sonda y al mismo nivel que el anterior, en el que se visualiza una sección axial de la VB con pared igualmente engrosada (*); c y d) Cuantificación del grosor mural vesicular (*) en un corte longitudinal paramedial del abdomen a nivel de hipocondrio derecho con sonda convex, en el que se observa una sección sagital de la VB con pared engrosada: c) 6,6 mm; d) 8,6 mm (normal < 3 mm). CF: cisura falciforme; LHD: lóbulo hepático derecho; LHI: lóbulo hepático izquierdo; R: riñón derecho; VCI: vena cava inferior; VP: vena porta.

**Figura 2:**
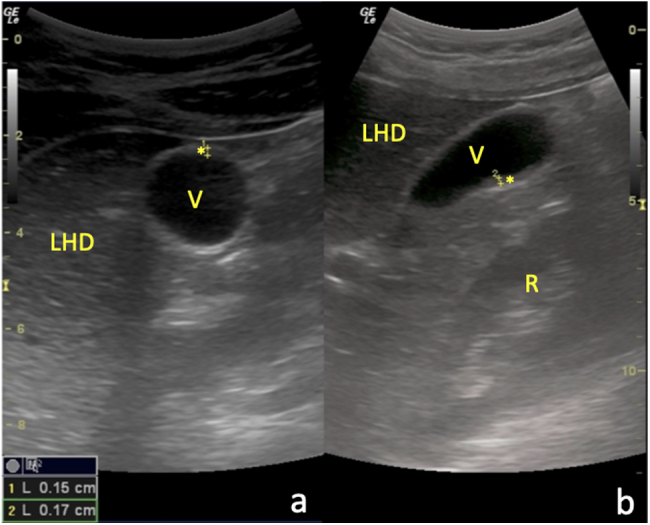
Resolución estructural de la CAA: a) Corte transversal del abdomen a nivel de hipocondrio derecho con sonda convex, en el que se objetiva una sección axial de la VB con pared de morfología y grosor normales (*); b) Corte longitudinal paramedial del abdomen con la misma sonda y al mismo nivel que el anterior, en el que se visualiza una sección sagital de la VB con pared igualmente normal (*). LHD: lóbulo hepático derecho; R: riñón derecho.

Este caso subraya la importancia de considerar la infección aguda por el VHA dentro del diagnóstico diferencial de la CAA en pacientes pediátricos. Asimismo, en niños diagnosticados con hepatitis A, debe mantenerse un alto índice de sospecha ante la aparición de síntomas abdominales, ya que el diagnóstico temprano de la CAA permite un abordaje no invasivo eficaz y previene la aparición de complicaciones^5^, ^6^.

## Financiación

Los autores manifiestan que no han recibido financiación alguna para la elaboración del manuscrito.

## Consideraciones éticas

Los autores confirman que se han obtenido todos los consentimientos requeridos por la legislación vigente para la publicación de cualquier dato personal o imágenes de pacientes, sujetos de investigación u otras personas que aparecen en los materiales enviados a Elsevier, se han realizado todos los procedimientos éticos y se han respetado los derechos de privacidad de los sujetos humanos.
